# Quality Characteristics and Inhibitory Xanthine Oxidase Potential of 21 Sour Cherry (*Prunus Cerasus* L.) Varieties Cultivated in China

**DOI:** 10.3389/fnut.2021.796294

**Published:** 2021-12-09

**Authors:** Rui Wang, Fang Zhang, Shengyue Zan, Chang Gao, Changping Tian, Xianghong Meng

**Affiliations:** ^1^College of Food Science and Engineering, Ocean University of China, Qingdao, China; ^2^Yantai Academy of Agricultural Sciences, Yantai, China

**Keywords:** sour cherries of china, physicochemical characteristics, new cultivars, correlation analysis, xanthine oxidase

## Abstract

This study aims to analyze the physicochemical characteristics and activities of 21 sour cherry cultivars in China. The evaluated accessions differ in several quality traits including weight, moisture, color, total soluble solids, and total acids. Glucose and malic acid were the predominant individual sugar and organic acid in all accessions. The potassium (K) and iron (Fe) were of the highest contents in Érid jubileum (453.887 mg/100 g FW) and Meili (2.988 mg/100 g FW), respectively. The contents of total phenolics (TP) were from 9.309 to 24.118 mg GAE/g DW, and total flavonoids (TF) were 8.935–27.198 mg RE/g DW, which were highly positively correlated (*r* = 0.892, *p* < 0.001). M-15, Érdi fubileum, and Érid jubileum showed the highest inhibitory effects on xanthine oxidase, and the IC_50_ inhibitory were 2.619, 3.117, and 3.940 mg/ml, respectively. This work evaluated the quality and nutritional characteristics of 21 sour cherry cultivars grown in China and explored their potential as an innovative food ingredient for hyperuricemia by evaluating the inhibitory effects of xanthine oxidase. And these results provide valuable data and new ideas for the future sour cherry breeding program as well as a processing guide.

## Introduction

Sour cherry, also called tart cherry, is native to Europe (1) and is popular in Europe and the United States. Compared with sweet cherries, sour cherry has its own characteristic astringent taste for higher acid/sugar ratios (2), is rich in polyphenolic compounds such as flavonoids (3), and has much higher amounts of hydroxycinnamic acids, procyanidins, flavonol glycosides, and flavanols (3–5). Hence, given the significantly high phytochemical contents, sour cherry is also branded as “Ultimate super-food” for its remarkable functions, such as antioxidant (6) and anti-inflammatory (7), especially the anti-gout effect (8, 9).

Uric acid is the final product of purine metabolism in humans. When the serum urate concentration was over 408 μmol/L (6.8 mg/dL), hyperuricemia is defined (10). In recent years, the prevalence of hyperuricemia and gout keep increasing dramatically, and the prevalence of hyperuricemia and gout in mainland China was 13.3% and 1.1%, respectively (11). Previous studies verified that reducing the activity of xanthine oxidase (XO), which is a key enzyme in purine metabolism, and inhibiting its catalytic oxidation of hypoxanthine to xanthine were both effective means to further inhibit uric acid (UA) production (12). It was found that polyphenolics have great potential for preventing diseases caused by the overproduction of radicals and the XO activity (12, 13). Because of no seasonal restrictions and ease of dose control, sour cherry provides a more convenient solution for the nutritional intervention of hyperuricemia. Therefore, sour cherry and its products are becoming more and more popular in China market.

As an introduced species, sour cherry is a new type of fruit in China, and some new breed sour cherry varieties have appeared and begun to be planted large-scale in Shannxi and Shangdong Province recently. However, the chemical compositions of sour cherries are of high variability among different cultivars (14) and different grown conditions (15). Therefore, it is necessary to analyze and compare the physical and chemical active components of sour cherry from different regions and strains.

Therefore, given the limited knowledge of Chinese cultivated sour cherry, the main purpose of this work was to characterize the physicochemical characteristics and nutrients. So, the fruit weight, moisture, and edible proportion were detected and calculated, and the contents of the total soluble solids, total acid, individual sugar, and organic acids were measured. And the nutrients such as potassium (K), iron (Fe), total phenolics, total flavonoids, and total anthocyanins were evaluated and analyzed. Aiming to explore their potential as an innovative food ingredient for hyperuricemia, the inhibitory effects of XO were evaluated. This work would help select the most valuable and desirable sour cherry cultivars in China.

## Materials and Methods

### Plant Materials

**Figure 1:**
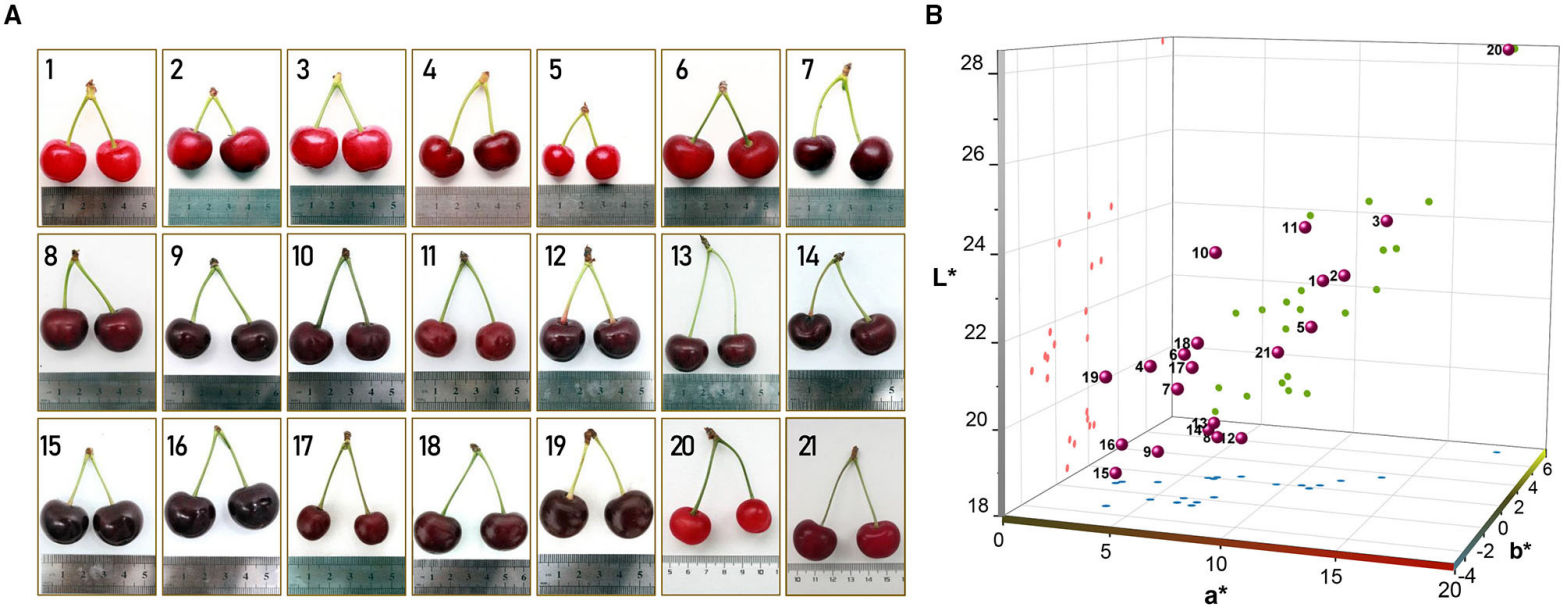
The cherry cultivars were used in the study. **(A)** the appearance of the 21 sour cherries; **(B)** the CIELAB color space diagram of the studied sour cherry fruit skins.

Twenty-one sour cherry varieties cultivated in China were studied in this work. “Meili”, “Meixue”, “Aode”, “Aojie”, and “Xiuyu” were the varieties bred by the Chinese researchers. The code names of the varieties given by the breeders were “zy-1”, “1–1”, “2–3”, “8–9”, “8–13”, “BS4”, “BS5”, “M-15”, and “H-6”. “Korosi early”, “Paraszt meggy”, “Érdi fubileum”, “Érid jubileum”, “Ujfehértoi fürbõs”, “Debreceni bõtermõ”, and “Earey hungazihn” were imported varieties. At the fully ripened stage, the fruits were randomly selected and harvested by hand (Table 1 and Figure 1).

**Table 1:** The information and quality traits of the 21 different sour cherry cultivars analyzed.

**No**.	**Names or Code names**	**Location**	**Altitude**	**Harvest date**	**Weight (g)**	**Edible proportion (%)**	**Moisture (g/100g)**	**TSS (°Brix)**	**TA(g malic acid/100g FW)**	**TSS/TA**
1	Meili	34.33°N,108.61°E	392 m	May 28, 2019	4.996 ± 0.033^e^	92.01 ± 0.11^de^	86.28 ± 0.14^ab^	14.1 ± 0.1^j^	1.980 ± 0.003^a^	7.12
2	Meixue	34.33°N,108.61°E	392 m	May 28, 2019	5.482 ± 0.029^c^	91.55 ± 0.25^de^	85.12 ± 0.1^bcd^	15.5 ± 0.1^h^	1.940 ± 0.016^ab^	7.99
3	Aode	34.33°N,108.61°E	392 m	May 28, 2019	5.678 ± 0.031^b^	92.6 ± 0.06^c^	87.91 ± 0.48^a^	15.5 ± 0.1^h^	1.957 ± 0.028^ab^	7.92
4	Aojie	34.33°N,108.61°E	392 m	May 28, 2019	5.724 ± 0.019^b^	93.07 ± 0.22^bc^	81.18 ± 0.44^gh^	18.1 ± 0.1^e^	1.499 ± 0.002^h^	12.07
5	zy-1	34.33°N,108.61°E	392 m	May 28, 2019	2.497 ± 0.012^n^	90.7 ± 0.41^f^	84.28 ± 0.81^bcde^	14.0 ± 0.1^j^	1.323 ± 0.001^j^	10.58
6	1–1	34.33°N,108.61°E	392 m	May 28, 2019	5.685 ± 0.019^b^	90.69 ± 0.26^f^	80.71 ± 0.32^hgi^	19.1 ± 0.1^c^	1.324 ± 0.002^j^	14.43
7	2–3	34.33°N,108.61°E	392 m	May 28, 2019	3.722 ± 0.02^j^	94.11 ± 0.09^a^	81.39 ± 0.84^fgh^	21.8 ± 0.1^a^	1.758 ± 0.003^d^	12.4
8	8–9	37.49°N,121.28°E	7 m	Jun. 14, 2019	5.079 ± 0.01^e^	93.27 ± 0.32^b^	82.34 ± 0.08^efg^	17.9 ± 0.1^ef^	1.319 ± 0.008^j^	13.57
9	8–13	37.49°N,121.28°E	7 m	Jun. 14, 2019	4.434 ± 0.186^h^	91.98 ± 0.09^de^	85.90 ± 0.41^abc^	14.8 ± 0.1^i^	1.255 ± 0.003^k^	11.79
10	BS 4	37.49°N,121.28°E	7 m	Jun. 14, 2019	3.234 ± 0.014^l^	90.44 ± 0.23^f^	84.67 ± 0.44^bcd^	16.3 ± 0.2^g^	1.541 ± 0.006^g^	10.58
11	BS 5	37.49°N,121.28°E	7 m	Jun. 14, 2019	2.495 ± 0.014^n^	90.32 ± 0.37^f^	85.86 ± 0.18^abc^	14.2 ± 0.1^j^	1.676 ± 0.017^ef^	8.47
12	M-15	37.49°N,121.28°E	7 m	Jun. 14, 2019	4.599 ± 0.054^g^	91.69 ± 0.03^de^	84.67 ± 0.63^bcd^	13.5 ± 0.5^k^	1.834 ± 0.008^c^	7.36
13	Korosi early	37.49°N,121.28°E	7 m	Jun. 14, 2019	3.364 ± 0.047^k^	90.24 ± 0.49^f^	83.25 ± 0.73^def^	19.9 ± 0.6^b^	1.922 ± 0.06^b^	10.35
14	Paraszt meggy	37.49°N,121.28°E	7 m	Jun. 14, 2019	4.289 ± 0.029^i^	91.42 ± 0.36^e^	85.12 ± 0.44^bcd^	16.1 ± 0.2^g^	1.765 ± 0.027^d^	9.12
15	Érdi fubileum	37.49°N,121.28°E	7 m	Jun. 14, 2019	4.308 ± 0.039^i^	93.03 ± 0.14^bc^	79.54 ± 0.05^hij^	19.9 ± 0.2^b^	1.780 ± 0.000^d^	11.18
16	Érid jubileum	37.49°N,121.28°E	7 m	Jun. 14, 2019	5.256 ± 0.031^d^	93.22 ± 0.21^b^	78.83 ± 0.54^ij^	18.5 ± 0.1^d^	1.649 ± 0.010^f^	11.22
17	Ujfehértoi fürbõs	37.49°N,121.28°E	7 m	Jun. 25, 2019	2.639 ± 0.019^m^	89.26 ± 0.23^g^	83.95 ± 0.22^cde^	17.6 ± 0.2^f^	1.053 ± 0.001^l^	16.71
18	Debreceni bõtermõ	37.49°N,121.28°E	7 m	Jun. 25, 2019	4.273 ± 0.024^i^	91.61 ± 0.34^de^	78.37 ± 0.43^j^	15.2 ± 0.1^h^	1.939 ± 0.023^ab^	7.84
19	Earey hungazihn	37.49°N,121.28°E	7 m	Jun. 25, 2019	5.890 ± 0.018^a^	93.55 ± 0.28^b^	84.83 ± 3.28^bcd^	14.9 ± 0.2^i^	1.394 ± 0.003^i^	10.69
20	Xiuyu	35.89°N,119.41°E	174 m	Jun. 6, 2020	4.755 ± 0.072^f^	93.36 ± 0.23^b^	87.12 ± 0.08^ab^	13.4 ± 0.2^k^	1.024 ± 0.011^l^	13.09
21	H-6	35.89°N,119.41°E	174 m	Jun. 6, 2020	4.714 ± 0.012^f^	92.04 ± 0.15^de^	85.22 ± 1.58^bcd^	14.2 ± 0.2^j^	1.696 ± 0.021^e^	8.37

*Values are expressed as mean ± standard. Cultivars sharing the same letter belong to the same subgroup, according to the Dunn's test (p < 0.05)*.

The fruits were delivered to the lab immediately after harvest and kept in the −80°C refrigerator (DW-86L486, Haier, Qingdao, China) for titratable acidity, reducing sugar, and minerals. The fruit mass, water content, the proportion of edible portions, and color were measured on the fresh berries. For the total phenolics, total flavonoids, total anthocyanins, antioxidant activity analyses, and xanthine oxidase inhibitory assay, samples without pit were freeze-dried for 48 h (FD5–5, SIM, L.A., USA) and crushed by mortar for homogeneous powder in liquid nitrogen. The powder was maintained at −80°C, waiting to be extracted and analyzed.

The extracts were prepared as below: the powder (1 g) was extracted using 60 ml of 80% aqueous methanol acidified with 1% formic acid at room temperature and stayed for 24 h in darkness. Then, the samples were filtered and transferred to a 100 ml volumetric flask filled with extractant to volume, and mixed.

### Chemicals and Reagents

Xanthine, Xanthine Oxidase, and Standards (rutin, malic acid, citric acid, tartaric acid, succinic acid, lactic acid, oxalate, fumarate, glucose, fructose, and sucrose) were purchased from Yuanye (Yuanye Bio-Tech, Shanghai, China). Single Element Standard Solution Fe (1,000 μg/ml) and K (100 μg/ml) were purchased from the National Center (Guobiao, Beijing, China). The acetonitrile and methanol for the high-performance liquid chromatography (HPLC) were from Merck (Merck KGaA, Darmstadt, Germany). The 2,2′-azinobis(3-ethylbenzothiaziline-6-sulfonate (ABTS) was obtained from Solabio (Solabio Sci&Tech, Beijing, China).

### Fruit Weight, Edible Proportion, and Moisture

Thirty berries of each cultivar were selected randomly. Their weights were evaluated by an electronic scale (BS124S, Sartorius, Göttingen, Germany). The cherry pits were removed and weighed, and the edible proportion was calculated as follows: Edible proportion (%) = (fruits weight - pits weight)/fruits weight × 100. The moisture was determined according to the method described by Schulz et al. (16).

### Color Measurement

The CIELAB scale was used for the fruit and juice color. The L^*^, a^*^, and b^*^ values were measured by the chroma meter (CR-400, Konica Minolta, Tokyo, Japan), according to the method described by Ly et al. (17). The chroma (C^*^) and hue (h^*^) can be calculated from the a^*^ and b^*^ values as: C^*^ = [(a^*^)^2^+(b^*^)^2^]^1/2^ and h^*^= arctan (b^*^/a^*^) (18).

### Total Soluble Solids and Titratable Acidity

The TSS was measured by the refractometric method (RX-5000α-Bev, ATAGO, USA). The titratable acidity was determined by titrating the diluted fruit juice to pH 8.20 with 0.1 mol/L NaOH (DELTA-320, Mettler Toledo, Zurich, Switzerland). And the TSS/TA ratio was calculated and used as an indicator of taste quality (19).

### Minerals

Cherry pulps of 1.0 g were weighed into digestion tubes and a digestion solution of nitric acid was added to digest until the samples were transparent or slightly yellow on the electric furnace. The samples were made up to 100 ml with ultrapure water and then diluted to a suitable concentration for analysis. The analysis of K was carried out by atomic absorption spectrophotometer (AA-6,800, Shimadzu, Kyoto, Japan) and Fe was analyzed by liquid chromatography inductively coupled plasma mass spectrometry (LC-ICP-MS/MS) (8,800, Agilent, Santa Clara, USA). To avoid interference, cesium chloride (0.2%) was added for K determinations.

### Total Phenolics, Total Flavonoids, and Total Anthocyanins

The TP, TF, and TAn content in the cherry extract samples were the basis of the method of Yan et al. (20). They were expressed as milligrams of gallic acid equivalents (GAE) per gram of dry weight (DW), milligrams of rutin equivalent (RE) per gram of DW, and milligrams cyanidin-3-glucoside equivalent (Cyd-3-Glu) per gram of DW, respectively.

### Quantification of Organic Acids and Individual Sugars

The organic acids were determined by the method described by Zhao et al. (21). Quantification was achieved using a calibration plot of external standards. The quantification was achieved using a calibration plot of external standards: malic acid (*y* = 3.2495*x* + 33.218; R^2^ =0.9995; concentrations of 12.5–200 μg/ml), citric acid (*y* = 1.7875*x* +1.3552; R^2^ =0.9998; concentrations of 8.0–129 μg/ml), lactic acid (*y* = 1.3525*x* + 2.2422; R^2^ =0.9999; concentrations of 33.3–533 μg/mL), tartaric acid (*y* = 3.4616*x* + 6.7397; R^2^ =0.9973; concentrations of 8.8–141.2 μg/ml), and fumaric acid (*y* = 284.69*x* + 6.8556; R^2^ =0.9999; concentrations of 6.2–100 μg/ml).

The quantitative analysis of individual sugars was carried out by HPLC (Chromaster, HITACHI, Tokyo, Japan) equipped with refractive index detector (RID) (5450, HITACHI, Tokyo, Japan) and were separated in Agilent ZORBAX Eclipse XDB-C18 (250 × 4.6 mm, 5 μm) (Santa Clara, USA) at 30°C. Eighty percent acetonitrile was used as mobile phase, and the flow rate was 0.8 ml/min. The standard curves (concentrations of 0.781–25.0 mg/ml) were glucose (*y* = 428741*x* – 447094; R^2^ =0.9990), fructose (*y* = 409423*x* - 229445; R^2^ = 0.9996), and sucrose (*y* = 391267*x* – 243540; R^2^ =0.9992).

### ABTS Tests

For the extracts, ABTS assays were used according to the method of Floegel et al. (22) described. The ABTS^·+^ scavenging activities were expressed as mM Vc equivalents (VCE) per gram of DW.

### Xanthine Oxidase Inhibitory Assay

The xanthine oxidase inhibitory assay was after the method described by Ahmed et al. (23). The extracts were evaporated to dry by the Hei-VAP Value (Heidolph, Schwabach, German). The extracts were screened for XO inhibitory activity at a final concentration of 20.00 mg/ml and diluted with phosphate-buffered saline (PBS) buffer to different concentrations. The reaction medium consisting of 60 μl of 1 mmol/L xanthine and 50 μl of the test sample was preincubated at 37°C for 5 min. To trigger the reaction, 30 μl of 0.02 unit/ml XO buffer solution was added. After incubation at 37°C for 30 min, the reaction was terminated by the addition of 50 μl of 0.5 M HCl. The absorbance at 295 nm was measured using a microplate reader (Powerwave XS, Biotek, Winooski, USA).

### Statistical Analysis

The data were recorded as mean ± SD of at least triplicate determinations. The Pearson's correlation coefficient was calculated by the Graphpad prism 9.0 (GraphPad Software Inc., California, USA). The significance levels for all tests were *p* < 0.05 and *p* < 0.01. SPSS 21.0 (IBM, Armonk, New York, USA) was adopted for the statistical study and analysis.

## Results and Discussion

The evaluated physical and chemical characteristics are presented in Tables 1, 2. These parameters, such as color, fruit weight, sugar, acid, and so on, are the critical varietal characteristic for consumption and processing, that may fluctuate depending on climate, soil, and agricultural conditions (14).

**Table 2:** The color parameters and nutrients of 21 sour cherry fruits.

**Names or**	**Skin color**					**Juice**	**Total phenolics**	**Total flavonoids**	**Total anthocyanins (mg Cyd-3-Glu/g DW)**	**K**	**Fe**
**Code Names**	L*	a*	b*	***C***	***H***	**color**	**(mg GAE/g DW)**	**(mg RE/g DW)**		**(mg/100 g FW)**	**(mg/100 g FW)s**
Meili	23.02 ± 1.01^bc^	11.40 ± 1.36^bc^	0.26 ± 0.59^cd^	11.41 ± 1.37^c^	180.02 ± 0.05^b^	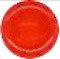	18.447 ± 0.566^d^	15.117 ± 0.391^e^	0.835 ± 0.054^j^	381.915 ± 5.064^de^	2.988 ± 0.010^a^
Meixue	23.08 ± 1.85^bcd^	12.08 ± 2.13^bc^	0.87 ± 1.13^bc^	12.15 ± 2.19^bc^	180.06 ± 0.09^b^	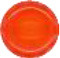	18.557 ± 0.451^d^	15.512 ± 0.503^ef^	0.599 ± 0.048^k^	332.696 ± 13.265^ghi^	2.234 ± 0.027^bcd^
Aode	24.37 ± 0.88^b^	13.71 ± 0.25^b^	1.55 ± 0.39^b^	13.81 ± 0.22^bc^	180.11 ± 0.03^b^	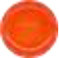	17.424 ± 0.308^ef^	15.105 ± 0.496^e^	0.612 ± 0.033^k^	449.854 ± 12.521^ab^	2.938 ± 0.115^ab^
Aojie	21.19 ± 0.67^e^	4.98 ± 2.92f^g^	−2.46 ± 0.98^i^	5.85 ± 2.34^fgh^	179.43 ± 0.33^f^	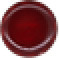	15.920 ± 0.129^gh^	11.353 ± 0.507^h^	3.056 ± 0.074^d^	427.757 ± 8.091^bc^	2.343 ± 0.102^abcd^
zy-1	21.95 ± 0.57^cde^	11.09 ± 1.37^c^	−0.12 ± 0.88^d^	11.11 ± 1.39^c^	179.98 ± 0.07^b^	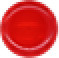	23.887 ± 0.327^a^	27.198 ± 0.916^a^	1.169 ± 0.030^i^	393.798 ± 2.261^d^	2.318 ± 0.329^abcd^
1–1	21.45 ± 0.63^de^	6.30 ± 1.25^ef^	−2.05 ± 0.44^hi^	6.67 ± 1.04^efg^	179.67 ± 0.13^cd^	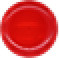	15.133 ± 0.135^h^	11.437 ± 0.155^h^	1.027 ± 0.032^i^	418.947 ± 9.043^c^	1.809 ± 0.117^d^
2–3	20.71 ± 0.34^ef^	6.28 ± 0.64^ef^	−2.48 ± 0.13^i^	6.76 ± 0.58^ef^	179.62 ± 0.05^ed^	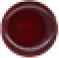	18.614 ± 0.34^d^	12.604 ± 0.043^g^	3.528 ± 0.129^c^	325.284 ± 27.850^hij^	2.151 ± 0.022^cd^
8–9	19.01 ± 0.74^g^	6.47 ± 1.21^ef^	0.09 ± 0.50^cd^	6.49 ± 1.23^efg^	180.00 ± 0.07^b^	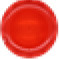	9.309 ± 0.219^j^	8.379 ± 0.216^i^	3.660 ± 0.130^bc^	304.899 ± 2.154^j^	1.950 ± 0.004^d^
8–13	18.76 ± 0.23^g^	4.19 ± 0.87^gh^	−0.88 ± 0.28^ef^	4.31 ± 0.80^hi^	179.77 ± 0.10^c^	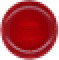	15.77 ± 0.965^gh^	13.550 ± 0.823^f^	3.592 ± 0.117^bc^	316.425 ± 11.433^ij^	2.251 ± 0.341^bcd^
BS 4	23.84 ± 4.00^b^	7.52 ± 1.04^de^	−1.72 ± 0.39^gh^	7.74 ± 0.95^de^	179.77 ± 0.07^c^	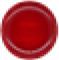	16.085 ± 0.753^gh^	15.957 ± 0.484^d^	2.257 ± 0.026^g^	343.943 ± 1.061^fgh^	0.919 ± 0.014e
BS 5	24.30 ± 0.89^b^	10.61 ± 0.57^c^	0.10 ± 0.41^cd^	10.62 ± 0.58^c^	180.01 ± 0.04^b^	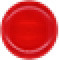	17.462 ± 0.854^ef^	15.264 ± 0.532^ef^	2.461 ± 0.008^f^	355.302 ± 19.018^fg^	1.990 ± 0.142^d^
M-15	18.97 ± 1.01^g^	7.48 ± 1.74^de^	0.37 ± 0.69^cd^	7.51 ± 1.75^def^	180.03 ± 0.10^b^	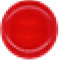	24.118 ± 0.859^a^	22.117 ± 0.421^b^	2.092 ± 0.138^h^	277.384 ± 0.044^k^	2.142 ± 0.175^cd^
Korosi early	19.40 ± 0.36^fg^	6.41 ± 1.13^ef^	−0.08 ± 0.36^d^	6.42 ± 1.11^efg^	179.98 ± 0.07^b^	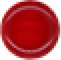	16.312 ± 0.341^g^	15.096 ± 0.293^e^	3.572 ± 0.075^bc^	331.435 ± 6.302^ghi^	2.786 ± 0.228^abc^
Paraszt meggy	19.21 ± 1.13^g^	6.11 ± 2.04^ef^	−0.04 ± 0.75^d^	6.14 ± 2.07^efg^	179.97 ± 0.10^b^	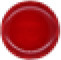	15.258 ± 0.214^h^	14.831 ± 0.084^e^	3.168 ± 0.065^d^	250.906 ± 2.938^l^	2.835 ± 1.385^abc^
Érdi fubileum	18.25 ± 0.76^g^	2.43 ± 0.30^h^	−1.33 ± 0.22^fg^	2.78 ± 0.24^i^	179.5 ± 0.10^ef^	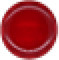	18.309 ± 0.834^de^	16.941 ± 0.561^c^	3.131 ± 0.203^d^	336.066 ± 4.565^fghi^	1.963 ± 0.039^d^
Érid jubileum	18.93 ± 0.28^g^	2.62 ± 0.52^h^	−1.17 ± 0.15^fg^	2.88 ± 0.42^i^	179.57 ± 0.12^de^	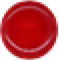	16.552 ± 0.747^fg^	13.384 ± 0.614^f^	2.480 ± 0.051^f^	453.887 ± 4.047^a^	2.217 ± 0.505^bcd^
Ujfehértoi fürbõs	21.27 ± 0.51^e^	7.05 ± 0.93^de^	−2.60 ± 0.18^i^	7.52 ± 0.87^def^	179.64 ± 0.05^cd^	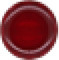	11.254 ± 0.691^i^	12.582 ± 0.199^g^	3.734 ± 0.189^b^	346.972 ± 14.042^fgh^	2.210 ± 0.004^bcd^
Debreceni bõtermõ	21.79 ± 0.28^cde^	7.09 ± 0.97^de^	−2.30 ± 0.36^hi^	7.48 ± 0.80^def^	179.68 ± 0.09^cd^	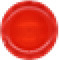	22.905 ± 0.527^b^	22.185 ± 0.354^b^	3.995 ± 0.063^a^	353.959 ± 4.885^fg^	2.122 ± 0.015^cd^
Earey hungazihn	21.05 ± 0.46^e^	3.54 ± 0.61^gh^	−3.36 ± 0.13^j^	4.90 ± 0.40g^h^	179.23 ± 0.10^g^	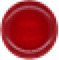	20.457 ± 0.501^c^	21.504 ± 0.351^b^	4.108 ± 0.134^a^	359.544 ± 0.473^f^	2.261 ± 0.297^bcd^
Xiuyu	28.41 ± 3.63^a^	17.81 ± 4.49^a^	5.20 ± 2.23^a^	18.55 ± 4.77^a^	180.28 ± 0.13^a^	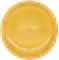	10.119 ± 0.197^j^	8.935 ± 0.226^i^	0.390 ± 0.003^l^	453.240 ± 21.883^a^	2.314 ± 0.235^abcd^
H-6	21.26 ± 0.70^e^	9.46 ± 2.91^d^	−0.03 ± 0.83^de^	9.48 ± 2.84^d^	179.99 ± 0.15^b^	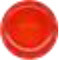	18.528 ± 0.463^d^	17.103 ± 0.303^c^	2.820 ± 0.036^e^	395.288 ± 0.091^d^	2.821 ± 0.140^abc^

*Values are expressed as mean ± standard. Cultivars sharing the same letter belong to the same subgroup, according to the Dunn's test (p < 0.05)*.

### The Physical Traits of the Sour Cherry Accessions Fruit Weight, Size, and Moisture

Fruit weight and size were shown in Table 1, both fruit weights and edible portions varied significantly (*p* < 0.05). The fruit weights ranged from 2.495 to 5.890 g, and their edible portions ranged from 89.26 to 94.11% (Table 1). The values are within the range of the studied sour cherries from other countries (15, 19, 24). Twelve of the investigated cultivars had larger fruits than the mean value of 4.434 g, lower than sweet cherries (25). Earey hungazihn, Aojie, 1–1, Aode, Meixue, 8–9, and Érid jubileum were characterized by the largest fruits (weight>5.0 g). In contrast, the weights of Ujfehértoi fürbõs, zy-1, and BS5 were lighter than 3.00 g, characterized by rather small fruits. Eleven of the edible parts of the cultivars were higher than the average value, over 91.91%. Ujfehértoi fürbõs was the only cultivar edible portion below 90%. The above-mentioned variability of parameters can influence the form of utilization and the technology of processing. Big fruits and high edible portions are favorable for both fresh consumption and processing acceptance (19, 24). Occasionally, smaller fruits may be preferred by processing companies for special products such as in chocolate or confectionery industries (19).

Moisture content is an important trait of fruit quantities and has a significant impact on other factors such as taste, texture, appearance, and weight. In this study, the moisture content values ranged from 78.37% (Debreceni bõtermõ) to 87.91% (Aode) (Table 1), which are in accordance with sweet cherries (from 75.08 to 88.56%) (25). The moisture content of China breeds Meili, Miexue, Aode, and Xiuyu were higher than the introduced cultivars, and this finding partly means that the China breeds were more suitable for juice processing.

### The Contents of TSS and TA in Sour Cherry Accessions

**Figure 2:**
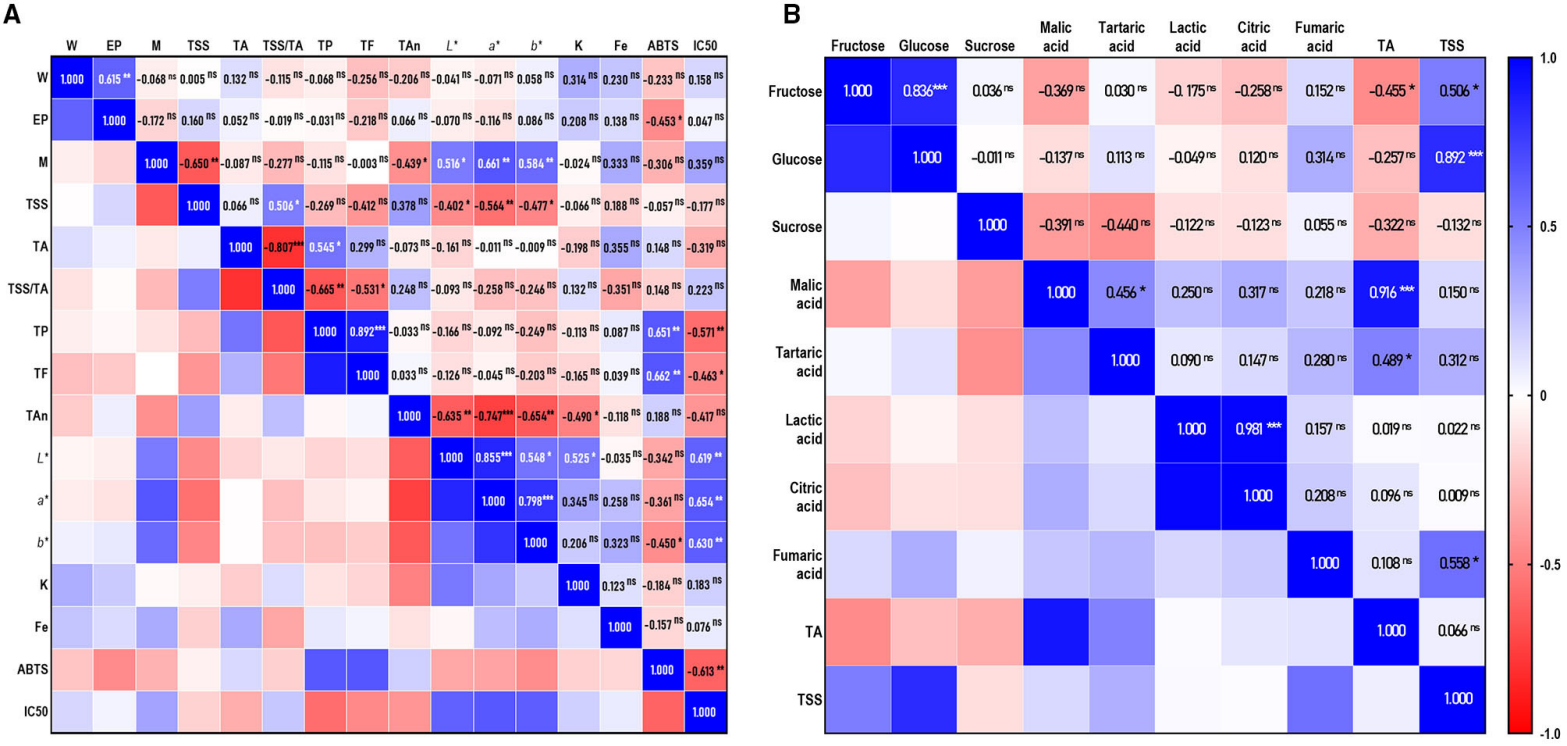
Correlation analysis of each trait. **(A)** Correlation coefficients among individual sugars, organic acids, total soluble solids (TSS), and titratable acid (TA); **(B)** Correlation coefficients between fruit traits and antioxidant properties. (*, **, *** Correlations significant at *p* < 0.05, *p* < 0.01, and *p* < 0.001. *ns* = not significant).

The TSS and TA are the two vital quality traits of fruits. Their ratio (TSS/TA), indicated by maturity, was a key factor responsible for the taste and flavor of fruits and influencing the preference and acceptance of consumers (14, 25). In this study, the TSS values varied from 13.4°Brix (Xiuyu) to 21.8°Brix (2, 3), significantly different (*p* < 0.05) among cultivars (Table 2), that was in the range of previewed work in German (19). Except Xiuyu and M-15, the TSS value of the other cultivars was all above 14.0, which was considered as a “desirable line” for sweet cherries (14). Because of the similar value of TSS with sweet cherries (14, 25), a higher value of TA was the reason for the lower TSS/TA in sour cherries (19), which resulted in a more sour taste of sour cherries. The sourest cultivars in this study were Meili, Aode, Meixue, and Debreceni bõtermõ, with the values of 1.980, 1.957, 1.940, and 1.939 g malic acid/100g FW, respectively. Xiuyu and Ujfehértoi fürbõs were the least acidic cultivars, with TA values of 1.024 and 1.053 g/100 FW. The TA values are in accordance with previously reported data, falling between 0.90 and 3.1 g malic acid/100g FW in Poland (24), Germany (19), and Croatia (26). While sweet cherries were low in acid with values between 0.40–1.5 g malic acid/100 g FW in Italy (27), Portuguese (25), Greece (14), and Iran (28). The values of the TSS and TA of sour cherries were similar with peaches (29) and pomegranates (30, 31). Moreover, according to Pearson's coefficient (Figure 2A), there is no correlation between TSS and TA, which may be caused by natural differences among cultivars (25, 27), and results in the difference from previous studies (*p* < 0.01, *r* = 0.597 and 0.42, respectively) (25, 28).

The TSS/TA ratio was considered as maturity index (MI), which classified fruits as sour, MI = 5–7; sour-sweet, MI: 17–24; and sweet, MI: 31–98 (29). The MI values of the tested accessions varied from 7.12 in Meili to 16.71 in Ujfehértoi fürbõs (Table 2). According to the classification, all the analyzed fruits were a sour-sweet taste rather than only a sour taste. And the values were higher than those of the sour cherries in Poland (15) and much lower than those of sweet cherries, which varied from 16.07 to 38.19 (25, 27). In particular, the MI values of Mieli, Miexue, Aode, Debreceni bõtermõ, and M-15 were near 7, closed to sour taste.

### The Individual Sugars and Organic Acids Profiling of Sour Cherries

In fruits, the most common sugars are glucose, fructose, and sucrose, which are responsible for sweetness perception, while the main organic acids responsible for sourness perception were usually malic acid, critic acid, tartaric acid, and so on. The contents of the individual sugars and organic acids are shown in Table 3. Glucose was the dominant sugar in the tested cultivars, followed by fructose and sucrose, which confirmed the previous researches in Turkey (32), Poland (15), Hungarian (33), Italy (27), and Greece (14). Ujfehértoi fürbõs, Érid jubileum, and Érdi fubileum had the highest glucose content, and Meili the lowest (12.239, 12.070, 10.475, and 6.399 g/100 g FW, respectively). The contents of fructose were from 3.008 to 7.054 g/100 g FW. The ratio of glucose/fructose was about two in all the invested cultivars, being consistent with the sour cherries in Turkey (32) as well as Hungarian (33), but different from that in Poland (the ratio about 1:1). By correlation analysis (Figure 2B), the content of glucose was highly correlated with TSS and fructose (r =0.892 and.836, respectively; *p* < 0.001), similar to the tendency described for cherries, including sour (33) and sweet cherries (27). The trace amounts of sucrose were identified in most cultivars, from 0.517 g/100g FW in Aode to 1.210 g/100g FW in Earey hungazihn, while absence in 1–1, Korosi early, and Debreceni bõtermõ.

**Table 3:** The organic acids and sugars of 21 sour cherry samples.

**Names or**	**Individual sugar (g/100 g FW)**			**Organic acid (mg/100 g FW)**				
**Code Names**	**Fructose**	**Glucose**	**Sucrose**	**Malic acid**	**Tartaric acid**	**Lactic acid**	**Citric acid**	**Fumaric acid**
Meili	3.008 ± 0.292^i^	6.399 ± 0.769^g^	0.804 ± 0.039^b^	1400.82 ± 35.91^abc^	54.13 ± 5.92^cdef^	275.92 ± 11.90^cdefgh^	209.27 ± 9.01^defgh^	0.11 ± 0.02^gh^
Meixue	3.641 ± 0.073^h^	8.076 ± 0.455^ef^	0.655 ± 0.005^b^	1432.41 ± 108.58^ab^	72.32 ± 52.52^f^	266.13 ± 98.13^defgh^	201.86 ± 74.25^defgh^	0.12 ± 0.04^gh^
Aode	3.653 ± 0.27^h^	7.780 ± 0.632^f^	0.517 ± 0.448^b^	1505.52 ± 145.18^ab^	98.41 ± 7.75^abc^	502.90 ± 69.87^ab^	381.01 ± 52.87^ab^	0.21 ± 0.03^efg^
Aojie	4.869 ± 0.247^def^	9.375 ± 0.667^c^	0.758 ± 0.045^b^	1238.51 ± 7.88^bcd^	66.09 ± 7.45^bcde^	446.52 ± 10.10^a^	338.35 ± 7.64^a^	0.43 ± 0.06^bc^
zy-1	4.476 ± 0.190^fg^	8.426 ± 0.207^cdef^	0.656 ± 0.014^b^	1077.03 ± 299.63^de^	59.55 ± 13.11^cdef^	412.28 ± 123.99^bcd^	312.44 ± 93.82^bcd^	0.12 ± 0.10^h^
1–1	5.757 ± 0.164^b^	11.288 ± 0.571^b^	-	1238.51 ± 7.88^bcd^	66.09 ± 7.45^bcdef^	446.52 ± 10.10^abc^	338.35 ± 7.64^abc^	0.43 ± 0.06^cd^
2–3	5.237 ± 0.255^cde^	11.252 ± 0.498^b^	0.744 ± 0.14^b^	1384.51 ± 56.09^abc^	61.42 ± 5.33^bcdef^	196.63 ± 45.52^efgh^	149.28 ± 34.44^fgh^	0.73 ± 0.01^a^
8–9	4.835 ± 0.173^efg^	9.148 ± 0.18^c^	0.743 ± 0.115^b^	1119.11 ± 130.57^de^	55.24 ± 2.08^cdef^	401.03 ± 204.76^bcd^	303.93 ± 154.93^bcd^	0.72 ± 0.12^a^
8–13	4.357 ± 0.183^g^	8.014 ± 0.503^def^	0.736 ± 0.059^b^	911.96 ± 23.75^ef^	59.24 ± 9.11^cdef^	159.75 ± 38.41^gh^	121.37 ± 29.06^gh^	-
BS 4	4.477 ± 0.537^g^	8.863 ± 0.59^cd^	0.797 ± 0.068^b^	1030.68 ± 37.16^de^	58.80 ± 4.16^cdef^	185.20 ± 29.10^fgh^	140.63 ± 22.02^fgh^	-
BS 5	4.404 ± 0.363^fg^	7.557 ± 1.064^ef^	0.831 ± 0.029^b^	1228.84 ± 6.15^bcd^	36.86 ± 0.03^f^	237.52 ± 21.31^defgh^	161.09 ± 10.93^efgh^	0.34 ± 0.14^de^
M-15	4.412 ± 0.406^fg^	7.499 ± 0.62^ef^	0.977 ± 0.12^ab^	1456.58 ± 161.01^ab^	63.31 ± 10.15^bcdef^	364.45 ± 20.55^bcde^	276.25 ± 15.55^bcde^	0.27 ± 0.09^ef^
Korosi early	5.399 ± 0.428^cde^	11.314 ± 1.016^b^	-	1387.00 ± 5.21^abc^	133.88 ± 0.41^a^	213.51 ± 2.25^efgh^	162.05 ± 1.70^efgh^	0.66 ± 0.01^ab^
Paraszt meggy	4.968 ± 0.388^efg^	9.377 ± 0.471^cd^	0.921 ± 0.081^b^	1461.61 ± 85.11^ab^	73.33 ± 9.62^bcdef^	326.11 ± 9.67^cdefg^	247.24 ± 7.31^cdefg^	-
Érdi fubileum	6.074 ± 0.508^b^	10.475 ± 2.5^ab^	0.712 ± 0.041^b^	1244.88 ± 32.5^bcd^	104.94 ± 7.17^ab^	156.41 ± 1.60^gh^	118.84 ± 1.21^h^	0.22 ± 0.01^efg^
Érid jubileum	6.829 ± 0.377^a^	12.070 ± 0.522^ab^	0.707 ± 0.019^b^	1255.73 ± 20.13^bcd^	50.52 ± 10.41^def^	287.29 ± 4.72^cdefgh^	217.87 ± 3.57^cdefgh^	-
Ujfehértoi fürbõs	7.054 ± 0.146^a^	12.239 ± 0.079^a^	0.756 ± 0.066^b^	850.65 ± 16.04^ef^	49.06 ± 4.11^ef^	294.92 ± 115.13^cdefgh^	139.06 ± 32.51^fgh^	0.11 ± 0.01^gh^
Debreceni bõtermõ	5.165 ± 0.435^cd^	8.354 ± 0.681^cdef^	-	1574.57 ± 359.34^a^	95.41 ± 55.91^abcd^	212.07 ± 5.47^efgh^	160.96 ± 4.14^efgh^	-
Earey hungazihn	5.215 ± 0.406^def^	9.121 ± 0.835^cde^	1.210 ± 0.449^a^	1060.17 ± 64.23^de^	54.63 ± 4.93^cdef^	124.32 ± 3.40^h^	94.56 ± 2.57^h^	0.23 ± 0^efg^
Xiuyu	5.666 ± 0.119^bc^	8.273 ± 0.728^cdef^	0.896 ± 0.136^ab^	707.54 ± 50.00^f^	-	164.27 ± 17.50^fgh^	124.79 ± 13.24^gh^	0.14 ± 0^fgh^
H-6	4.995 ± 0.348^cde^	7.616 ± 0.272^f^	0.919 ± 0.213^ab^	1213.82 ± 94.2^bcd^	85.90 ± 6.77^bcde^	341.71 ± 28.93^bcdef^	259.05 ± 21.89^bcdef^	0.65 ± 0.14^ab^

*Values are expressed as mean ± standard. Cultivars sharing the same letter belong to the same subgroup, according to the Dunn's test (p < 0.05)*.

The predominant organic acid in all the accessions was malic acid, with highly positively correlated with TA that was described above (*r* = 0.916; *p* < 0.001) (Figure 2B). The content of organic acids differed widely among the cultivars: the contents of malic acid ranged from 707.54 (Xiuyu) to 1,574.57 mg/100 g FW (Debreceni bõtermõ), accounting for 52.12 to 72.48% of the total organic acids; lactic acid ranged from 124.32 (Earey hungazihn) to 583.96 mg/100 g FW (Aojie), accounting for 9.32–25.03% of the total organic acids; citric acids ranged from 94.56 (Earey hungazihn) to 442.35 mg/100 g FW (Aojie), accounting for 7.09–18.96% of the total organic acids; trace amount of fumaric acid was detected in most cultivars.

### The Color Traits of the Sour Cherry Accessions

Color plays an important role in consumer acceptance and is one of the most important features for a product with high quality (14, 34). The color characteristics of the sour cherries are shown in Table 2. For a more intuitive comparison of skin colors, the CIELAB color space diagram (Figure 1B) was made by L^*^, a^*^, and b^*^ values, which stand for lightness, redness, and yellowness (17). Results were shown that the L^*^, a^*^, and b^*^ values were significantly different, and Xiuyu was the most different one among the accessions, with the highest L^*^, a^*^, and b^*^ values (28.41, 17.81, and 5.20) and a golden color juice. On the whole, the obtained L^*^ values varying from 18.95 to 43.30 and a^*^ value from 1.9 to 33.5, which are corroborated with previous studies in sweet cherries, while negative b^*^ values were obtained in most accessions, that was very different from sweet cherries (14, 25, 27). It was worth noticing that the darker cultivars tended to be less red and less yellow (Figure 1B), such as Érdi fubileum, Érid jubileum, Paraszt meggy, Korosi early, 8–9. Furthermore, these cultivars showed lower values of chrome (*C*) and hue angle (*H*), corresponding to a darker and more intense color. The differences among sour cherry cultivars in fruit color may also underline the variability in the accumulation pattern of specific anthocyanin molecules (14), the larger the number of hydroxyl groups on the B-ring, the bluer the color (35). And as pH increase, the color of anthocyanin moves to the non-spectral purple and approaches a progressive loss of fruit color. This could partially explain the difference in color traits L^*^, a^*^, and b^*^ along with the variances of TA (Figure 2A).

### The Contents of TP, TF, and TAn in the Sour Cherry Accessions

Phenolics were the main secondary metabolites of sour cherries, associated with various capabilities. The total phenolics, flavonoids, and anthocyanins were detected in this study. The results are shown in Table 2. The contents of TP ranged from 9.309 ± 0.219 mg GAE/g DW in 8–9 to 24.118 ± 0.859 mg GAE/g DW in M-15 and the contents of TF were ranged from 8.935 ± 0.226 mg RE/g DW in Xiuyu to 27.198 ± 0.916 mg RE/g DW in zy-1. The significant differences are also shown in TAn contents of the accessions. Debreceni bõtermõ and Earey hungazihn had the highest amount of TAn (3.995 and 4.108 mg Cyd-3-Glu/g DW, respectively), and Xiuyu had the lowest content (0.390 mg Cyd-3-Glu/g DW). M-15, zy-1, Debreceni bõtermõ, and Earey hungazihn showed the highest level of TA and TF in the accessions. Pearson correlation analysis (Figure 2A) also confirmed that the content of TP and TF was of significantly positive correlation (r=0.892, *p* < 0.001). However, neither of them correlated with the content of anthocyanins. Both TP and TAn contents of most accessions were higher than that of Montmorency (10,323±1,468 and 482±56 μg/g DW) and Balaton (7,752±932 and 1,063±178 μg/g DW) in America (5).

### Mineral Traits of the Sour Cherry Accessions

Cherries were considered to be a good source of dietary K, which played an important role in the Dietary Approaches to Stop Hypertension (DASH), and could reduce blood pressure and stroke risk (1), as well as lowering serum urate among gout patients (36). In the accessions, the contents of K ranged from 250.906 to 453.887 mg/100 g FW, much higher than the records in the USDA MyPyramid nutrient data 173 and 222 mg/100 g FW at sour and sweet cherries respectively (1). From the Correlation analysis (Figure 2B), the contents of K showed moderate relationships to total anthocyanins (*r* = −0.490, *p* < 0.05) and L^*^ (*r* = 0.595, *p* < 0.05). According to the literature, the high K application rate affected the L^*^ value (37) and reduced anthocyanins (38), which indicated that K might be related to fruit color. In this regard, the fruit K contents variation could partly explain the detected differences in the color variances.

Iron is also an important microelement for human health. The Fe contents of the accessions were ranged from 0.919 mg/100 g FW to 2.988 mg/100 g FW, and they were 5.4–17.6 times higher than sour cherries with USDA recorded 0.17 mg/100 g and their products (6). The lowest content of Fe was 0.919 mg/100 g FW detected in BS 4, significantly lower than the other accessions (1.809–2.988 mg/100 g FW at 1–1 and Meili), which were closed with wild cherries (the range from 1.8 to 3.96 mg/100 g FW) (6).

### ABTS Free Radical Scavenging Capacities

**Figure 3:**
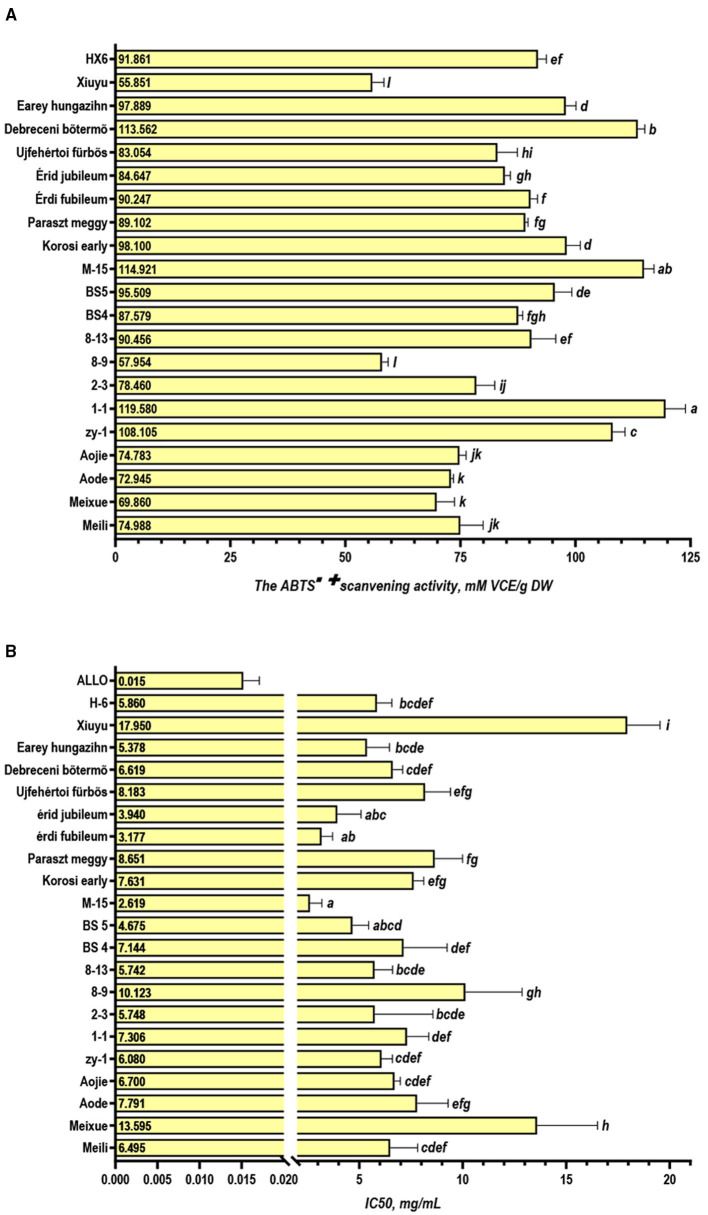
Activity analysis of sour cherry extract. **(A)** The ABTS^·+^ scavenging activity; **(B)** IC_50_ for xanthine oxidase inhibitory activity of sour cherry extracts (*p* < 0.05).

The ABTS assay was one of the most sensitive and speedy methods applied as the commonly antioxidant assay methods (39). It applied to both hydrophilic and lipophilic antioxidant systems (40) and performed better with high-pigmented and hydrophilic antioxidants (22). Because 80% methanol aqueous solution was used as the extraction agent, the extracts were a hydrophilic material rich in pigment. Hence the ABTS assay was adopted for antioxidant capability in this study. The results (Figure 3) showed that 1–1, M-15, and Debreceni bõtermõ displayed the highest activity (119.580, 114.912, and 113.562 mM VCE/g DW, respectively), while Xiuyu and 8–9 were the lowest with 55.851 and 57.954 mM VCE/g DW, respectively. Furthermore, we found that the ABTS scanning capability was positively relative to the total phenolics and flavonoids (r =0.651 and r =0.662, *p* < 0.01) (Figure 2A). This correlation was consistent with the conventional knowledge, that the antioxidant capacity is highly correlated with the phenolic content (22). Through Pearson correlation analysis, a negative correlation was found between the scavenging ability of ABTS and IC_50_ of XO (*r* = −0.613, *p* < 0.01) (Figure 2A), which might be also related to the content of phenolics.

### Xanthine Oxidase Inhibitory Activity

Inhibiting XO is one of the most effective methods for reducing the amount of serum UA (41). In this study, 50% inhibitive concentration (IC_50_) for the xanthine oxidase inhibitory activity of sour cherry extracts were detected and the results are shown in Figure 3B. The xanthine oxidase inhibitory activity observed with all accessions suggested that sour cherries contained a substantial amount of phytoconstituents with xanthine oxidase inhibitory potential. Among them, the extracts of M-15 displayed obvious activity with IC_50_ of 2.619 mg/ml, followed by Érdi fubileum (3.177 mg/ml) and Érid jubileum (3.940 mg/ml), while Xiuyu and Meixue displayed very weak inhibitory activities with the IC_50_ of 17.950, and 13.595 mg/ml, respectively. Meili, Aojie, and Aode displayed a moderate activity with the IC_50_ of 6.495, 7.700, and 7.791 mg/ml, respectively. Many results indicate that polyphenolics have a great potential for preventing diseases caused by XO activity (13, 23, 39). Therefore, we furtherly studied the relationship among TP, TF, TAn, and the IC_50_ of XO. The results showed that the Pearson correlation analysis also revealed a moderate correlation between the IC_50_ of XO and TP (*r* = −0.571, *p* < 0.01), TF (*r* = −0.463, *p* < 0.05), but no correlation with TAn (*r* = −0.417, ns) (Figure 2A). The results suggested that polyphenolics and flavonoids, rather than anthocyanins, were the important components in the inhibition of XO activity in the extract of sour cherries. The substitution of different groups on varied positions of flavonoid scaffolds greatly influenced the binding behavior of compounds on the active site of XO, resulting in the difference in the inhibition activity (13).

Xanthin oxidant (XO) inhibitors, allopurinol and febuxostat, are currently used as first-line clinical drugs in many countries (10, 42). But there are many side effects such as hepatitis, nephropathy, and allergic reaction (12, 13), that limited their use. And for patients with asymptomatic hyperuricemia, experts agreed that lifestyle advice on diet, weight loss, or exercise would be applied rather than drug treatment (42). Thus, many researchers were focused on searching for natural XO inhibitors with less toxic and side effects (12, 13, 23). Many clinical studies have found that sour cherry can reduce the content of UA and delay the onset of gout (8, 9). And the results of this work also proved that sour cherry extract has good XO inhibition ability, which indicates the potential application of sour cherry in the treatment and daily management of patients with gout in the future.

## Conclusions

In this study, fruit quality traits, nutrients, and the inhibitory effect on the XO of 21 sour cherry cultivars grown in China were analyzed. The predominant sugar and organic acids were glucose and malic acid. All the tested sour cherries showed a high level of K and Fe content, and sour cherries in China could be a good plant-based source of K and Fe. Meili, Meixue, Aode, Korosi early, and Debreceni bõtermõ showed a high level of TA. Regarding the phenolic levels, zy-1, M-15, Debreceni bõtermõ, and Earey hungazihn possessed higher amounts of total phenolics, flavonoids, and anthocyanins. All the tested cultivars showed inhibition of XO, but the inhibition ability was different among cultivars, which were significantly stronger in M-15, Debreceni bõtermõ, and Earey hungazihn, relating to the high content of phenolics and flavonoids. Moreover, the Chinese cultivars, Meili, Meixue, Aode, and Aojie were more suitable for processing, because of their characteristics of big size, high moisture, and high acids. While Xiuyu was more promising for fresh eating due to the low acids and high-water content. Overall, our findings provide valuable data and new ideas for the future sour cherry breeding program.

## Data Availability Statement

The raw data supporting the conclusions of this article will be made available by the authors, without undue reservation.

## Author Contributions

RW: writing–original draft. FZ: funding acquisition, writing–review and editing. SZ: methodology and formal analysis. CG: supervision and data analysis. CT: provided the concept. XM: provided the concept. All authors contributed to the article and approved the submitted version.

## Funding

This work was supported by the National Natural Science Foundation of China (32172275), Natural Science Foundation of Shandong Province (ZR2019BC049), and China Postdoctoral Science Foundation (2018M642705).

## Conflict of Interest

The authors declare that the research was conducted in the absence of any commercial or financial relationships that could be construed as a potential conflict of interest.

## Publisher's Note

All claims expressed in this article are solely those of the authors and do not necessarily represent those of their affiliated organizations, or those of the publisher, the editors and the reviewers. Any product that may be evaluated in this article, or claim that may be made by its manufacturer, is not guaranteed or endorsed by the publisher.

## Abbreviations

ABTS: 2,2′-azinobis(3-ethylbenzothiaziline-6-sulfonate)
C: Chrome
Cyd-3-Glu: Cyanidin-3-glucoside equivalent
DASH: Dietary Approaches to Stop Hypertension
DW: Dry weight
FW: Fresh weight
GAE: Gallic acid equivalents
H: Hue angle
RE: Rutin equivalent
TAn: Total anthocyanins
TA: Titratable acidity
TF: Total flavonoids
TP: Total Phenolics
TSS: Total soluble solid
UA: Uric acid
VCE: Vc equivalents
XO: Xanthin oxidant.
